# Outcome of Laparoscopic Cholecystectomy at a Secondary Level of Care in Saudi Arabia

**DOI:** 10.4103/1319-3767.74484

**Published:** 2011

**Authors:** Abdulrahman S. Al-Mulhim, Tarek T. Amin

**Affiliations:** Department of Surgery, College of Medicine at Al –Ahsa, King Faisal University - Al –Ahsa, Saudi Arabia; 1 Family and Community Medicine Department, College of Medicine at Al –Ahsa, King Faisal University - Al –Ahsa, Saudi Arabia

**Keywords:** Complications, laparoscopic cholecystectomy, morbidity

## Abstract

**Background/Aim:**

The first option for gallbladder surgery is laparoscopic cholecystectomy. The aim of this study is to analyze the outcomes for all patients who underwent laparoscopic cholecystectomy at a secondary level of care.

**Patients and Methods:**

Between 2005 and 2008, 968 consecutive laparoscopic cholecystectomies were performed at King Fahad Hospital. We collected and analyzed data including age, gender, body mass index (kg/m^2^), the American Society of Anesthesiologists (ASA) class, mode of admission (elective or emergency), indication for LC (chronic or acute cholecystitis [AC]), co-morbid disease, previous abdominal surgery, conversion to open cholecystectomy, complications, operation time, and length of postoperative hospital stay.

**Results:**

Nine hundred and sixty-eight patients had laparoscopic cholecystectomy at the center. There were 824 females and 144 males; the age range was 15-64 (mean 32.9± 12.7 years). The operating time was 45 to 180 min (median 85 min); the complication rate was 4.03% (39 patients).

**Conclusion:**

Laparoscopic cholecystectomy could be performed safely in the majority of patients with cholelithiasis, by an experienced surgical team at a secondary level of care.

Surgeon Muhe from Germany (1985), and surgeon Mouret from France (1987), performed the first human laparoscopic cholecystectomy (LC) cases.[1,2]

Epidemiological studies indicate that 70% to 80% of all cholecystectomy are now completed laparoscopically, and it is one of the most commonly undertaken procedures in general surgery.[3]

Several clinical and epidemiological studies suggest that the outcome of LC depends on factors such as age, gender, body weight, clinical presentation, previous abdominal surgery, and surgeon’s experience.[4]

However, morbidity and mortality rates are usually used to evaluate the outcome in a surgery.[5,6]

The aim of this study was to assess the outcome of LC at the secondary level of care in terms of morbidity.

## PATIENTS AND METHODS

This is a record-based descriptive study carried out in Surgical Department at King Fahd Hospital over a period of 4 years from 1 January 2005 to 31 December 2008. King Fahad Hospital at Hofuf is located in Al Hassa Governorate, Eastern Saudi Arabia, 350 km from Riyadh, with a population of about 1 million people. The hospital provides secondary level of care and represents the main referral center for diagnostic and surgical procedures in Al Hassa.

All records of patients of both genders and of those above the age of 12 years (< 12 years is considered pediatric in our hospital) who underwent LC irrespective of its indications were included in this study.

The collected data included age, gender, body mass index (kg/m^2^), American Society of Anesthesiologists (ASA) class, mode of admission (elective or emergency), indication for LC, co-morbid disease, previous abdominal surgery, diagnostic investigations, duration of the procedure, hospital stay, and complications.

Patient selection for surgery was made preoperatively based on history, physical, and laboratory diagnostic evidence of gall bladder disease.

Admitted patients were required to undergo the standard pre-operative tests liver function test, renal function tests, screening for hepatitis, blood sugar, and complete blood picture.

Ultrasonogram was routinely performed on all patients to confirm the clinical diagnosis of cholelithiasis with number of stones, sizes, gall-bladder wall thickness, and pericholecystic collection, and diameter of common bile duct.

Hypertension, diabetes mellitus, bronchial asthma, and cardiac disease were the most common co-morbid diseases found in this series.

LC was performed using the standard four-port technique advocated by Reddick;[6] the pneumo-peritoneum was created by the closed method, and diathermy of the gallbladder was performed with the monopolar electrosurgical hook in all cases.

During this study period, four consultant surgeons with vast experience in open surgery performed LC.

### Data processing and analysis

Collected data were analyzed using the statistical package for social sciences (*SPSS, version* 13.0; Chicago, IL, USA). Categorical data were expressed in frequency and percentage; numerical data were expressed in medians, mean, and standard deviations. Univariate analysis and chi-square test were employed to determine possible correlates of the patient outcome and time along the period of study. Binary logistic regression analysis was generated by inclusion of significant variables at the univariate level to assess potential predictors of LC outcomes (occurrence of complications) in relation to patient’s characteristics and pre-operative morbidity encountered taking the occurrence of operative and postoperative complications including conversion as the dependent variable.

### Ethical considerations

Data confidentiality was maintained during all phases of data collection and analysis.

## RESULTS

This hospital records-based study included 968 patients.

This study was carried out during the period 2005 to 2008, females represented nearly 85% of cases (female to male ratio of 5.8:1), the age of the included patients ranged from 15 to 64 years (mean 32.9± 12.7 years). Those in the age group of 20 to <40 years represented the main bulk of patients who underwent LC [Table 1].

**Table 1:** Basic and clinical preoperative patient characteristics

Characteristics	Year				Total (N=968) No. (%)
	2005 (N=255) No. (%)	2006 (N=263) No. (%)	2007 (N=271) No. (%)	2008 (N=179) No. (%)	
Gender					
Female	216 (84.7)	223 (84.8)	233 (86.0)	152 (84.9)	824 (85.1)
Male	39 (15.3)	40 (15.2)	38 (14.0)	27 (15.1)	144 (14.9)
Age group					
< 20 years	13 (5.1)	15 (5.7)	18 (6.6)	9 (5.0)	55 (5.7)
20- < 40	141 (55.3)	145 (55.1)	149 (55.0)	99 (55.3)	534 (55.2)
40- <50	76 (29.8)	79 (30.0)	81 (29.9)	54 (30.2)	290 (30.0)
≥ 50	25 (9.8)	24 (9.1)	23 (8.5)	17 (9.5)	89 (9.2)
Co-morbidity†					
Diabetes	45 (17.6)	52 (19.8)	56 (20.7)	27 (15.1)	180 (18.6)
Hypertension	47 (18.4)	49 (18.6)	50 (18.8)	29 (16.2)	175 (18.1)
Coronary heart	10 (3.9)	7 (2.7)	23 (8.5)	7 (3.9)	33 (3.4)
Respiratory problems	3 (1.2)	2 (0.8)	-	2 (1.1)	7 (0.7)
Hemolytic blood disease	8 (3.1)	8 (3.0)	9 (3.3)	11 (6.1)	40 (4.1)
Previous abdominal surgery	3 (1.2)	2 (0.8)	0 (0)	2 (1.1)	7 (0.7)
Body mass index					
Desirable (BMI<25)	176 (69.0)	167 (63.5)	174 (64.2)	111 (62.0)	628 (64.9)
Overweight (BMI 25-30)/Obese (BMI>30)	79 (31.0)	96 (36.5)	97 (35.8)	68 (38.0)	340 (35.1)
Gall bladder stones	61 (23.9)	66 (25.1)	44 (16.2)	44 (24.6)	242 (25.0)
Single					
Multiple	191 (74.9)	193 (73.4)	198 (73.1)	133 (74.3)	715 (73.9)
Gall bladder poly.	3 (1.2)	4 (1.5)	2 (0.7)	2 (1.1)	11 (1.1)
Common bile duct diameter					
< 5 mm	168 (65.9)	175 (66.5)	173 (63.8)	121 (67.6)	637 (65.8)
≥ 5 mm:	87 (34.1)	88 (33.5)	90 (36.2)	58 (32.4)	323 (34.2)
History of cholangitis	5	3	4	3	
History of pancreatitis	7	3	3	2	
Obstructive jaundice and ERCP	11	6	5	4	

† Not mutually exclusive, ERCP: Endoscopic retrograde cholangiopancreatography, BMI: Body mass index

All patients were assigned an American Society of Anesthesiology (ASA) physical status classification: 513(53%) were ASA I, 442 (45.7%) were ASA II, and 13 (1.3%) was ASA III.

Table 2 shows the pre-operative status of the gall bladder and complications encountered following LC. Intra-operatively in 355(36.7%) patients, there was flimsy adhesion of the gallbladder. In 406(41.9%), gallbladder wall was found to be thickened (chronic cases), whereas in another 96(9.9%) cases gallbladder wall was inflamed (acute cholecystitis).

**Table 2:** Peri-operative status of the gall bladder and encountered complications following laparoscopic cholecystectomy (year 2006-2008)

Variables	Year				Total (N=968) No. (%)
	2005 (N=255) No. (%)	2006 (N=263) No. (%)	2007 (N=271) No. (%)	2008 (N=179) No. (%)	
Peri-operative status					
Adhesions	93 (36.5)	97 (36.9)	99 (36.5)	66 (36.9)	355 (36.7)
Gall bladder status					
Acute inflammation	22 (8.6)	27 (10.3)	31 (11.4)	16 (8.9)	96 (9.9)
Chronic inflammation	107 (42.0)	110 (41.8)	115 (42.4)	74 (41.3)	406 (41.9)
Gangrenous bladder	1 (0.4)	1 (0.4)	3 (0.7)	1 (0.6)	6 (0.6)
Mucocele	7 (2.7)	8 (3.0)	10 (3.7)	6 (3.4)	31 (3.2)
Normal	118 (46.3)	121 (46.0)	112 (41.3)	82 (45.8)	433 (44.7)
Operative time (minutes)					
< 60	31 (12.2)	39 (14.8)	46 (17.0)	29 (16.2)	145 (14.9)
60- <120	166 (65.1)	179 (68.1)	179 (66.1)	121 (67.6)	645 (66.6)
> 120	58 (22.7)	53 (20.1)	46 (16.9)	29 (16.2)	186 (19.2)*
Post-operative complications					
Minor					
Wound hematoma	2 (0.8)	1 (0.4)	-	-	3 (0.31)
Atelectasis	1 (0.39)	1 (0.38)	-	2 (1.1)	4 (0.41)
Wound infections	7 (2.7)	2 (0.8)	3 (1.1)	4 (2.2)	16 (1.7)
Epigastric port site hernia	-	2 (0.8)	-	1 (0.6)	3 (0.31)
Major					
Bile leak	-	-	2 (0.8)	1 (0.6)	3 (0.31)
Collection in pouch of Morrison	3 (1.1)	2 (0.8)	-	-	5 (1.9)
Conversion to open	3 (1.1)	2 (0.8)	-	-	5 (1.9)
More than one complications	4 (1.6)	3 (1.1)	2 (0.7)	2 (1.1)	11 (1.1)
Total cases with complications	16 (6.3)	10 (3.8)	5 (1.8)	8 (4.5)	39 (4.03)
Hospital length of stay	2 (1-13)	1 (1-11)	2 (1-12)	1 (1-9)	2 (1-13)
Median (range) in days					

* Statistically signifi cant Chi-square for trend

Distended gallbladders with mucocele were found in 31 (3.2%) patients which were decompressed laparoscopically.

Selective preoperative endoscopic retrograde cholangiopancreatography (ERCP) was performed in 26 cases because of obstructive jaundice, and a dilated common bile duct on ultrasound. In 19 cases in whom choledocholithiasis were present, it was managed with preoperative ERCP and endoscopic sphincterotomy [Table 1].

The operating time, from the time of insertion of the Verees needle to the end of skin closure of puncture wounds, ranged from 45 to 180 min (median 85 minutes).

Conversion from laparoscopic to open cholecystectomy was necessary in five cases. The reasons for conversion to open cholecystectomy were due to acutely inflamed and edematous gallbladder in two cases, bleeding from cystic artery in one case, and in two patients due to unclear anatomy of the operative field.

Postoperative bile leakage occurred in three patients (0.31%). All these patients had drains placed at the time of surgery. In two patients, it stopped on the fifth postoperative day, and one patient who continued to drain bile was successfully managed by endoscopic sphincterotomy and stent insertion.

In five patients, with acute cholecystitis and obscure anatomy, there were collections in Morrison’s pouch. It was irrigation saline. All cases were successfully managed by non-surgical measures.

The follow up period ranged from 6 months to 40 months (median 28 months), and no late complications were detected.

## DISCUSSION

LC is one of the most commonly undertaken procedures in general surgery since its inception in the early 1990s with low morbidity and mortality.[7] Large series of LC were reported with few complications.

In this study, pre-operative co-morbidities were found in 281 patients (29.0%). Type 2 diabetes was the most prevalent (18.6%); diabetes and hypertension were reported in 118 (12.2%), hypertension alone in 57 patients, coronary heart disease was found in 3.4% (of which 28 patients have had diabetes and hypertension), respiratory problems in the form of chronic asthma in5 patients, and obstructive diseases were also reported. This is similar to that reported by others.[8,9]

Table 1 shows the pattern of pre-operative; co-morbidities did not show significant trend in relation to the time period included.

Out of the 968 patients included, 340 patients (35.1%) were either overweight or obese based on their calculated body mass index (BMI), females constituted 67.1% of them. Obesity had no influence on the outcome of LC in this study, and this result is comparable to other studies that show no influence of BMI on the complications of LC.[8–10]

Table 2 displays pre-operative status of gall bladder and the frequency of encountered complications, during the period of study. Among patients, multiple gall bladder stones were common (73.9%) than single stone, and common bile dilatation (>10 mm) reported in nine patients.

Obscure anatomy and adhesions were found in 36.7% of cases, acute cholecystitis in 9.9% of cases, and gangrenous gall bladder was found in 0.6%.

Wound infections and hematoma were the most common complications encountered post-operatively. The complication rate was 4.03% (39 patients) with insignificant trend in relation to year of operation.

The 1.9% overall conversion rate among the study patients is similar to that reported by others.[10,11]

The operative time in minutes ranged from 45 to 180 min (median 85 min) and showed a significant trend over time as improvement was noticed in the shortening of operative time from year 2005 to 2008 (*P*= 0.047).

Hospital length of stay ranged from 1 to 13 days (for non-complicated cases a median of 2 days with a range of 1 to 3 days), while for complicated cases a minimum of four[4] and maximum of 13 days were reported.

**Figure 1:**
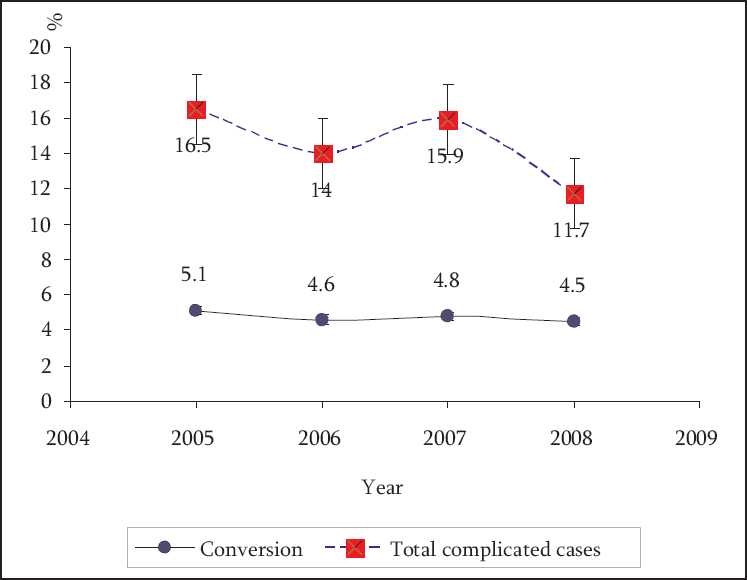
Total percentages of all complications encountered and the rate of conversion to open operation in relation to year of operation

Figure 1 demonstrates the total percentages of all complications encountered and the rate of conversion to open operation in relation to year of operation. Complications and conversion rates showed some pattern (although it was consistent) of downward trend in relation to time but these trends were statistically insignificant.

Table 3 demonstrates both univariate and multivariate binary logistic regression analyses. Female gender, the presence of co-morbidities, and multiple bladder stones, although increased the risk of complications, were found statistically insignificant at the univariate analysis.

**Table 3:** Univariate and multivariate analyses of determinants for laparoscopic cholecystectomy post-operative complications including conversion

Variables	Complications		Univariate odds ratio (95% C.I)	Multivariate logistic regression		
	Yes (N=39) No. (%)	None (N=929) No. (%)		B coeffi cient	Odds ratio (95% C.I)	*P* value
Gender						
Males	8 (20.5)	129 (13.9)	ref.			
Females	31 (79.5)	800 (86.1)	1.59 (0.98-2.55)	-	-	-
Co-morbidity						
No	24 (61.5)	501 (53.9)	ref.			
Yes	15 (38.5)	428 (46.1)	1.35 (0.92-1.97)	-	-	-
Age groups						
< 40	14 (35.9)	599 (64.5)	ref.			
≥ 40	25 (64.1)	330 (35.5)	2.85 (1.97-4.15))**	0.382	1.63 (1.03-2.58)	0.011*
Body mass index						
Desirable (BMI<25)	16 (41)	640 (68.9)	ref.			
Overweight (BMI 25-30)/	23 (59)	289 (31.1)	3.11 (2.13-4.56))**	0.551	1.78 (1.32-2.39)	0.030*
Obese BMI>30						
Gall stones						
Single	9 (23.1)	232 (25)	ref.			
Multiple	30 (76.9)	697 (75)	1.39 (0.91-2.14)	-	-	-
Bile duct diameter						
< 5 mm	15 (38.5)	651 (70.1)	ref.			
≥ 5 mm	24 (61.5)	278 (29.9)	3.76 (2.56-5.54))**	0.719	1.81 (1.43-2.29)	0.001*
Adhesions						
No	15 (38.5)	623 (67.1)	ref.			
Yes	24 (61.5)	306 (32.9)	2.87 (1.96-4.19))**	0.310	1.65 (1.22-2.24)	0.003*
Gall bladder status						
Normal	8 (20.5)	437 (47)	ref.			
Acute/gangrenous/mucocele	23 (59)	78 (8.4)	3.20 (1.97-5.20)**	0.210	1.39 (0.83-2.35)	0.080
Chronic	8 (20.5)	414 (44.6)	1.39 (0.95-2.01)	0.096	1.11 (0.78-1.58)	0.641

Ref. = reference group

* statistically signifi cant

** *P* value = 0.001. C.I = Confi dence intervals. For logistic regression model a constant was 1.637, Chi-square = 33.481, *P* = 0.001, percent predicted = 76.3

Patients of older age (≥ 40 years), obese and overweight, dilated bile duct (include patients with history of cholangitis, pancreatitis, obstructive jaundice), presence of adhesions, and with acute bladder inflammation were found to be significant predictors to complications and unfavorable outcome during univariate analysis.

Binary logistic regression found that age of the patient (Odds ratio (OR) = 1.63), obesity (OR= 1.78), presence of dilated common bile duct (OR= 1.81), presence of adhesions (OR= 1.65), were all positive predictors for the development of post-operative complications and were included with patients who underwent LC.

The status of gall bladder failed to appear as a significant predictor in the logistic regression model although it was significant at the univariate analysis.

Epigastric port site hernias developed in three patients who were diagnosed within the first 6 months post-operatively.

The outcomes of this case series of LC performed at a secondary level of care were equivalent to those in the surgical literature from tertiary care settings.[2,8,11–14]

## CONCLUSIONS

The results of this study confirm that LC at a secondary level of care in Saudi Arabia is a safe and effective intervention in selected patients with symptomatic gallstones, as in major teaching hospitals in Saudi Arabia.[9]

## References

[1] Reynolds W (2001). The first laparoscopic cholecystectomy. JSLS.

[2] Mouret P (1990). Celioscopic surgery: Evolution or revolution?. Chirurgie.

[3] Livingston EH, Rege RV (2004). A nationwide study of conversion from laparoscopic to open cholecystectomy. Am J Surg.

[4] Simopoulos C, Botaitis S, Polychronidis A, Tripsiani G, Karayiannakis AJ (2005). Risk factors for conversion of laparoscopic cholecystectomy to open cholecystectomy. Surg Endosc.

[5] Russell EM, Bruce J, Krukowski ZH (2003). Systematic review of the quality of surgical mortality monitoring. Br J Surg.

[6] Martin RC 2nd, Brennan MF, Jaques DP (2002). Quality of complication reporting in the surgical literature. Ann Surg.

[7] Reddick EJ (1992). Laparoscopic cholecystectomy in freestanding outpatient centers. J Laparoendosc Surg.

[8] Osborne DA, Alexander G, Boe B, Zervos EE (2006). Laparoscopic cholecystectomy: Past present, future. Surg Technol Int.

[9] Al-Mulhim AA (2008). Male gender is not a risk factor for the outcome of laparoscopic cholecystectomy: A single surgeon experience. Saudi J Gastroenterol.

[10] Angrisani L, Lorenzo M, De Palma G, Sivero L, Catanzano C, Tesauro B (1995). Laparoscopic cholecystectomy in obese patients compared with nonobese patients. Surg Laparosc Endosc.

[11] Bittner R (2006). Laparoscopic surgery: 15 years after clinical introduction. World J Surg.

[12] Giger UF, Michel JM, Optiz I, Th Inderbitzin D, Kocher T, Krahenbühl L (2006). Risk factors for perioperative complications in patients undergoing laparoscopic cholecystectomy: Analysis of 22,953 consecutive cases from the Swiss Association of Laparoscopic and Thoracoscopic Surgery database. J Am Coll Surg.

[13] Salam IM, Own A, Kareem NA, Hameed OA, Yak CJ, Zaki KA (2005). Laparoscopic cholecystectomy in the Academy Medical Centre, Khartoum, Sudan. East Afr Med J.

[14] Rather GM, Ravi VK (1997). Audit of laparoscopic cholecystectomies in a district general hospital. Saudi J Gastroenterol.

